# Practical Medicine

**Published:** 1881-02

**Authors:** 


### PRACTICAL MEDICINE.

LACTIC ACID IN CHRONIC CYSTITIS. (Deecke, *Revue de Thér. Méd-Chir* and *L' Union Méd. du Can*, Oct., 1880. Page 447.)

Of all the acids which Dr. Deecke has tried in the treatment of chronic catarrh of the bladder, lactic acid appears to him to be the most efficacious, and to give the most durable results. His formula is as follows: lactic acid, 1 to 2 grams, sweetened water q. s. Dissolve. To be taken three times a day. The sweetened water may be replaced by butter-milk or a bitter infusion. The lactic acid is found in the urine after the ingestion of three or four grams. It arrests rapidly the ammoniacal decomposition of urine in the bladder as well as outside of this organ, dissolves the salts which abound there, destroys the microscopic vegetables which develop there, and in consequence acts efficaciously upon the catarrh of chronic cystitis.

CHLORAL AS AN ANÆSTHETIC FOR CHILDREN. (M. Rédier. *L'Union Médicale du Canada*, October, 1880. Page 459.)

The author remarks that children enjoy a peculiar tolerance of chloral by which they are able to bear four or five grams several days in succession, while the same dose would be badly supported by adults.

The doses, which appear necessary and sufficient to produce anæsthesia, are: from two to four years, 2 grams; from four to eight years, 3 grams; from eight to twelve years, 4 grams. The best mode of administration is a potion of 100 grams of equal parts of water and syrup of gooseberries taken at one dose fasting. It is best not to proceed to an operation (such as an extraction of a tooth, opening of an abscess, etc.) for an hour or an hour and a half after the beginning of the sleep, the anæsthesia being more complete after a certain time. The duration of the sleep is from four to five hours.

Chloral has been employed as an anæsthetic for long and painful operations, such as the correction of vicious attitudes and ankyloses (Bouchut); and even for an operation for hare-lip in a child of six years, to whom 2½ grams of chloral had been given.

NOCTURNAL TERRORS IN CHILDREN. (Steiner. *Jour. de Méd.*, Sept., 1880. Page 414.)

The child generally awakes suddenly from one to three hours after going to sleep, with symptoms of the greatest terror; the face is agitated, the heart beats violently, the pulse is animated; not recognizing anything around, it remains deaf to the reassuring exhortations lavished upon it. Such is the expression of terrible anxiety it manifests, that all its senses appear to be riveted to the impression produced upon it by some terrifying image; this tableau lasts a quarter of an hour, twenty minutes and even more; then the child becomes a little calmer, recognizes the persons around it and gradually falls asleep again under the influence of the reassuring words it hears, to awake in the morning lively and well, and unable to remember what has happened during the night. These nocturnal attacks present many different degrees as to violence and duration not only in different children, but also in the same child at different times; thus it may happen that the child awakes with a start, utters some loud unintelligible sounds, stares around with a frightened look, trembles slightly with its hands and feet, and in two or three minutes falls asleep, after which follows profuse sweating; in other cases the attack lasts an hour or more. The attack is often followed by copious evacuation of urine destitute of qualitative alterations. Rarely there are two or three attacks in a single night; in these cases ordinarily the fits are mild; the paroxysms are repeated some times in following nights, but generally there are intervals of weeks or even months. In none of his cases did Steiner discover convulsions.

Most of Steiner's cases were among children from three to six years of age, consequently in subjects past the period of dentition; worms could not be accused of being the cause, nor could gastric troubles, such as diarrhœa, etc. These cases were nearly all in children who were pale and anæmic or rachitic and scrofulous of which they bore the marks; added to this there was an exaggerated nervous excitability to which it is logical to relate the described manifestations.

The prognosis in the majority of cases is favorable; by improving the general nutrition and bringing to bear suitable physical and moral treatment, the trouble will disappear without leaving any traces. It is only when the paroxysms come on frequently and violently that this complex of symptoms should be regarded as the precursor of a grave cerebral malady and be treated as such.

The state of the general nutrition, of the physical and psychical education of the child should be especially inquired into. All sorts of emotions should be proscribed, and especially terrifying emotions, emanating from exciting stories calculated to inflame the imagination of children. In case of frequent returns of the attack, the use of bromide of potassium associated with a feeble dose of hydrate of chloral at night, is particularly recommended (after West).

MORBID SWEATING. M. Bouveret, (*Jour. de Méd.*, Sept., 1880, p. 408.)

M. Bouveret, who has just been nominated *professeur agrégé* of the Faculty at Lyons, after a remarkable concours, has given a high degree of interest to the difficult subject treated in his thesis for the position. We mention a few of the most interesting points practically.

He mentions some very remarkable cases of ephidrosis or local sweating, generally but little known, but which are nevertheless important. For example, sweating of the legs, mentioned by Verneuil as a frequent sign of profound varices. There is a notable and habitual increase of the sweat, which is often accompanied by itching, eczema and erythema; the fact is important to note in the diagnosis of deep varicose veins which is often difficult.

Another singular example of local sweating is parotidian ephidrosis; it occupies quite exactly the region of the parotid gland, is not continuous, but generally intermittent and only appears at the time of eating, during mastication. The sweating sometimes extends beyond the parotid region, and is seen on the neighboring parts and even over a considerable portion of the face. In all the cases an injury is met with at the beginning of the trouble, a wound or opened abscess of the parotid gland. The duct of Steno is most often closed, but the phenomenon is not due to a transudation of the saliva as one would suppose. It is really a local sweating of reflex origin. Like that of the parotid region the facial ephidrosis is often reflex, and due to excitation of the nerves of taste.

The hypersecretion sometimes extends over the entire face, sometimes it remains unilateral; it has been seen exactly limited to the region of the face supplied by the superior maxillary branch of the trigeminal. DeGraefe has seen four cases of palpebral ephidrosis. The skin of the eyelid presented a well marked hyperæmia, and on this red spot, when examined by a lens, one could see, in case of an effort or an emotion, a clear fluid escape from a multitude of little orifices. Ephidrosis finally may occupy one entire side of the body.

An habitual general hyperidrosis or hypersecretion may show itself in variable etiological conditions generally but little known, but among these hyperidroses, one of the most interesting is that of the menopause, which M. Liégeois has just studied very completely. It is a fact well known that at the epoch of the menopause, women are subject to flashes of heat and sweating; but what is less known is, that these sweats, though independent of any other affection may become morbid. It may not be the case always of women who no longer have their courses; the hyperidrosis may come earlier and appear at that period when the near approach of the menopause announces itself already by certain irregularities of menstruation. It is not always a passing convenience; M. Liégeois cites several cases where women were affected by it for several years; he was able, however, in most cases, to check it by the use of atropine. In most of these patients the hyperidrosis appeared especially towards the end of the night. M. Liégeois advises the administration of atropine some hours before the expected return of the sweating. Half a milligram is a sufficient dose; it is well to continue the medicine several days after the cure.

Beside these different forms of morbid sweats, M. Bouveret has studied those which are characterized by a peculiar color. Chromidrosis, for example, or blue sweat, so rare that its existence has been denied by many authors, is nevertheless a scientific fact. Like sweating of blood, this singular alteration of the sweat appears most often among that set of symptoms which characterizes the neuropathic state, hysteria. Frequently violent moral emotion is the occasional cause of it; nearly always the eyelid is first attacked, and by preference the lower one; the blue sweat may however appear in other regions, the feet, the axilla, the epigastrium, the forehead, the cheeks; the ears are always spared. Sometimes the blue coloration extends over large surfaces; sometimes it is developed in little patches of the integument. The color varies from blue to black, passing sometimes to deep violet. The abnormal secretion proceeds by successive attacks, returning at variable intervals, generally preceded, as in the first attack, by disorders of the menstruation, by local derangements of the circulation or of sensibility. The coloring matter which gives the special aspect to this secretion is analogous to indigo, and its secretion seems to be produced under the influence of a vasomotor trouble.

The same is true of hematidrosis, a phenomenon very much discussed and of which the labors of M. Parrot especially have clearly shown the nature.

The sweating of blood which shows itself nearly always in hysterical women, may occupy very variable extents of the integument, sometimes oozing out in droplets, sometimes in the form of filiform jets; the liquid is composed of blood containing all its elements. It is intermittent, proceeds by steps, coincident with painful eruptions of the skin. These hæmorrhages are never alarming from their abundance; moreover the fluxes of blood in hysteria often enjoy the singular privilege of not compromising the health or the life by reason of their quantity for the hematidrosis should be regarded as a sort of hæmorrhagic hysteria, all the more as it accompanies, in many cases, hæmorrhages of the stomach, of the uterus, of the bronchia, of which the neuropathic nature can not be doubted; its existence, on the contrary, is doubtful except in hysteria.

We cannot dwell longer upon the different parts of the interesting work of M. Bouveret; we mention only that one chapter is consecrated to treatment in which the remarkable and nearly constant effects of atropine, administered according to the method of M. Vulpian, are clearly demonstrated. It is also shown that in a good many diseases, far from respecting profuse sweats as a useful symptom, on the contrary, one should combat them; this should be done especially in acute articular rheumatism, where the abundant sweats may be suppressed without inconvenience.

HYDROPHOBIA. (Bouley. *Hygiène pour tous*, and *L'Union Méd. du Canada.*) Oct., 1880, p. 448.

On the occasion of a very interesting report of a case of hydrophobia, by Prof. Haveley to the *Acad. de Médecine*, M. Bouley, inspector general of the veterinary schools of France, formulated this great truth:

" *The best way to prevent attacks of this redoubtable malady is to understand it.*"

This knowledge, the readers of *Hygiene for All* will have the good fortune to owe to M. Bouley himself, who has kindly authorized our editor-in-chief to reproduce the *résumé* of his work on *Hydrophobia, the means of avoiding its dangers, and of preventing its propagation.*

Here is the tableau of the characters of hydrophobia, as the eminent professor of the museum has so ably indicated them.

I. The madness of the dog is not characterized by fits of fury in the earlier days of this manifestation. On the contrary, it is a disease of benign appearance at first; but from its beginning, the saliva is virulent, that is, it contains the inoculable germ, and the dog is then much more dangerous from the caresses of its tongue than it may be from its bites, for it has as yet no tendency to bite.

II. At the beginning of its madness, the dog changes its temper; it becomes sad, sober and taciturn, seeks solitude and retires into the most obscure corners. But it cannot remain long in one place; it is unquiet and agitated, comes and goes, lies down and gets up, roams around, smells, hunts about, scratches with its fore feet. Its movements, its attitudes and its gestures seem to indicate that, for the moment, it sees phantoms, for it bites the air, springs forward and howls as if it were attacking real enemies.

III. Its look is changed; it expresses a sombre sadness and something of the ferocious.

IV. But in this state the dog is not at all aggressive toward man; its character is what it was before. It is docile and submissive to its master, whose voice it obeys, showing some signs of gaiety which bring back, for an instant, the usual expression to its physiognomy.

V. Instead of aggressive tendencies, they are often the contrary, which are manifested in the first stage of madness. Affectionate sentiments toward its masters and familiars about the house are exaggerated in the mad dog, and it expresses them by repeated movements with its tongue, with which it is eager to lick the hands or faces of those it can reach.

VI. This sentiment, very highly developed and very tenacious in the dog, dominates it so much, that in many cases it respects its masters, even in a paroxysm of rage, and so much that they, moreover, preserve great control over it, even when its ferocious instincts commence to manifest themselves and the dog abandons itself to them.

VII. The mad dog has not a dread of water; on the contrary, it is eager for it. As long as it can drink, it satisfies its thirst which is always great; and when spasm of its fauces prevents its swallowing, it plunges its nose into the water and bites, so to say, the liquid which it is unable to swallow. The mad dog then is not *hydrophobic; hydrophobia* is not then a sign of madness in the dog.

VIII. The mad dog does not refuse its food in the first period of its malady; often even it eats more voraciously than usual.

IX. When the desire to bite, which is one of the essential characteristics of the disease at a certain stage of the development, begins to manifest itself, the animal satisfies it at first upon inert bodies. It gnaws the wood of doors and of furniture, tears clothes, carpets, shoes, grinds straw, hay, hair, wool, between its teeth, eats the ground, and the dung of animals, even its own etc., and accumulates in its stomach the dêbris of everything with which its teeth have come in contact.

X. The abundance of the saliva is not a constant sign in the mad dog. Sometimes the mouth is moist, sometimes dry. Before the period of fits, the secretion of saliva is normal; it is exaggerated during this period and dries up at the end of the disease.

XI. The mad dog often expresses the painful sensation it feels from spasm of the fauces, by making with its forefeet on each side of its cheeks the peculiar gestures which a dog does in whose throat a bone is lodged.

XII. In a peculiar variety of canine madness which is called dumb madness, the paralyzed lower jaw is separated from the upper one, and the mouth remains open and dry with a brownish red color of the mucous membrane which covers it.

XIII. In some cases the rabid dog vomits blood which comes, in all probability, from wounds of the stomach caused by the sharp bodies which it has swallowed.

XIV. The voice of the rabid dog always changes in *timbre* and its bark is always completely different from its usual manner. It is rough, husky and is transformed into a jerky howl. In the variety called dumb madness this important symptom is lacking. The disease receives its name from the absolute dumbness of the patients; *dumb or mute madness.*

XV. The sensibility is very much diminished in the mad dog. When struck, or burnt, or wounded, it utters no complaints, nor cries by which animals of his species express their sufferings or even their fears simply. There are cases where the rabid dog makes deep wounds upon itself with its teeth, and assuages its rage upon its own body, without seeking to injure the persons with which it is familiar.

XVI. The mad dog is always very violently impressed and irritated by the sight of an animal of its own species. When it finds itself in the presence of one or hears its barking, its rabid fury is manifested, if it was still latent, or develops and increases if it was already declared, and it springs toward it to tear it with its teeth. The presence of the dog produces the same impression on animals of other species when they are under the influence of rabies; so that it is correct to say that the dog fills the office of a reactive agent, by the aid of which one may nearly always, with very great surety, bring out the madness still concealed in an animal which has it.

XVII. The rabid dog often leaves its home at the moment when, in the progress of its malady, ferocious instincts develop in it and begin to dominate over it; and after one, two or three days of wandering, during which it has sought to satisfy its rage upon all living beings which it has encountered, it frequently returns home to die with its master.

XVIII. When the madness has arrived at its period of fury, it is characterized by the impression of ferocity which it gives to the physiognomy of the affected animal, and by the desire to bite which it satisfies every time an occasion for it is presented; but it is always against one of its species that it directs its attacks by preference to any other animal.

XIX. The rabid fury is manifested by fits, in the intervals between which the animal, exhausted, falls into a state of relative calm which may cause a doubt as to the nature of its malady.

XX. Healthy dogs seem to be endowed with the faculty of divining the rabid state of an animal of their species, and, instead of quarreling with it, they seek to escape its attacks by flight.

XXI. The rabid dog when free, attacks first, and with great energy, all living beings it may meet with, but always by preference a dog rather than other animals, and these rather than man. Then, when it has exhausted its fury by its attacks, it goes forward with a vacillating gait, with tail pendant, head inclined toward the ground, its eyes looking wild, and mouth open, from which escapes the bluish tongue covered with dust. In this state it does not have very aggressive tendencies, but it still bites everything, man or beast, that comes in its way.

XXII. The mad dog which dies naturally succumbs to paralysis and asphyxia. Up to the last moment, the instinct to bite controls it, and it should be feared even when exhaustion seems to have transformed it into an inert body.

XXIII. At the autopsy of a rabid dog there is nearly always seen in the stomach, a conglomeration of incongruous bodies, such as hay, straw, hairs, wool, shreds of cloth, pieces of leather, remnants of twine, tow, excrements, earth, leaves, grass, stones, everything which by their presence togather, have a great probative value of the existence of rabies in the animal in whom found.

XXIV. The surest means of preventing the effects of rabic inoculation is immediate cauterization, preferably with the red-hot iron, or lacking this, gunpowder or caustic agents. The sooner this cauterization is made the more one can count on its efficacy.

XXV. If the cauterization cannot be made immediately after the bite, one should, while waiting, wash out the wound, press very energetically to squeeze out the blood, sucking the wound, rejecting very quickly the fluid drawn by the mouth, compress very tightly its edges continually, and apply if possible a circular ligature to arrest the circulation of the blood.

ON THE PRESENCE OF CERTAIN PROPORTION OF PRUSSIC ACID IN THE SMOKE OF TOBACCO, AND THE EXISTENCE OF A NEW ALKALOID. By Le Bon. (*J. de Therap., Sept. and Oct.,* 1880.)

The smoke of tobacco contains nicotina, carbonate of ammonium, various tarry substances, coloring matters, prussic acid combined with bases, aromatic principles strongly odoriferous and very toxic, aqueous vapor, various gaseous compounds, especially oxide of carbon and carbonic acid (carbonic anhydrid).

The toxic properties of tobacco smoke do not depend merely on nicotina, but on prussic acid, several aromatic principles, on a special alkaloid, *collidine*, a liquid with an agreeable odor, strong, and imparting to the smoke its peculiar odor, as much deleterious as nicotina.

But the vertigo, headaches and nausea, caused by various tobacco samples lacking in nicotina, are owing to prussic acid and various aromatic principles. Nicotina is not destroyed by the combustion of tobacco, and can be found largely in the smoke. The smoke of tobacco contains about 8 liters (17 lbs.) oxide of carbon for a 100 gr. (22 lbs.) of smoked tobacco; but the toxic qualities of tobacco are not owing to this gas as some German writers held.

The most certain results on man of the smoke of tobacco are visual troubles, palpitation, a tendency to vertigo, and specially an impaired memory.

DR. VIART read a paper at the academic seance of November 9, on The Treatment of Diphtheria. It is thus recapitulated:

1. Diphtheria (*angine couennense*) is a disease local at first, and most often becoming general only after the fourth or sixth day. A certain set of symptoms characterize it: *i. e.*, a brisk appearance of a false membrane in the throat, without pain nor any general reaction; a peculiar course, and the curability of the disease before it has become general. In twenty-six cases in which the throat was cauterized after the removal of the false membrane, Dr. Viart obtained twenty-six cures.

2. The parts through which the disease makes its entrance are most always the free surface of the tonsils; yet, in children, it may start in the larynx, producing croup, which is very rare in the adult.

3. *Course.*—It is divided in two periods: the first extending to the sixth day, during which the disease can be destroyed *in situ*—it is the curable period; the second extends from the sixth to the tenth or twelfth day; the disease has been absorbed in the organism—it is the stage of danger.

4. This being agreed upon, it is wise to intervene, as soon as the disease is diagnosed, with an energetic local treatment and general treatment also; during the dangerous stage, an exclusively general treatment has to be relied upon; yet if it is not evident that the constitution is already affected, cauterization should be had recourse to even then, since it could do no harm.

5. The local treatment advocated by Dr. Viart, consists in the violent (harsh) destruction of the false membrane, with the index finger, wrapped in a piece of linen, and pushed into the pharynx with some amount of friction, and a cauterization of the bleeding surface with nitrate of silver. This treatment should also be accompanied by the use of chlorate of potassium internally and topically, the use of alcoholics, and a sustaining diet.

### MENTAL STATE OF THE EPILEPTIC.

There are 40,000 epileptics in France, of which number 4,000 are in asylums, and 36,000 at large. Trousseau said, in 1855: "This is the disease most generally overlooked." There are several classes of epileptics: 1. The true epileptics; 2. Giddy epileptics; 3. Mental (larvés) epileptics; 4. Alcoholic epileptics. We will treat of the first three catagories.

1. *Epileptics, properly so-called.*—There are three varieties of them: (*a.*) Those whose intellect is not impaired; (*b.*) Those whose intellect is impaired momentarily; (*c.*) Truly insane epileptics.

In the first, most interesting and least studied class, the patient remains intelligent; nay, some may have their mental power unusually developed. Famous instances are on record: Julius Cæsar, Petrarc, Newton, and Molière himself, seemed to belong with these.

The epileptic who commits a crime, obviously is not always irresponsible; before rendering a judgment in the matter, one must inquire into the mental state of the patient, whether he has more or less persisting troubles of the intellect.

2. *Giddy epileptics.*—After a simple vertigo, they may commit the most inexplicable crimes. There is a suspension of the function of the cerebrum lasting five or six seconds; a *between-the-acts* in their existence; after this is over, the epileptic often ends the sentence which he had begun before the attack, and is unconscious of the latter.

Following the crisis, the patient may utter nasty expressions, or he may have momentary cerebral disorders. It is hard to persuade law courts that a man in this condition may unconsciously do something immoral. This is called absent mindedness, an appropriate term. It is in such circumstances that some epileptics steal from the counters, even in the presence of guardians; others scald their hands in boiling water.

Trousseau had noticed two phases of epilepsy only: the giddiness, and the fit. Herpin, Voisin, Legrand du Saulle, have described the incomplete fit; an intermediary state; the patient stops, turns his head toward one side, his face becomes expressionless, red, and the muscles are contracted; the patient produces motions of deglutition; there is no initial yell, nor fall. The incomplete fit lasts from fifteen to twenty seconds; patients call it false fits; it is a prelude to a greater fit.

A patient, having had an incomplete fit, does not remember the facts; he thinks that he witnessed something horrible. A typical sign is, an unceasing repetition of the same word, peculiar to epilepsy alone; at times this may be obscene; it may take place during the night only.

Epileptics' impulsions are well known. These are mental acts consisting in an impulse to commit thoughtless actions. This is observed besides in hereditary mental diseases. It is quick, imperious, thoughtless, impressive; a kind of mental convulsion which is controlled by bromide of potassium.

True epileptics, giddy epileptics with partial attacks, are given to anger, to egotism ; they love nobody, make it their aim to molest others, and are incapable of a pleasant action. They sometimes have an instinctive wish to run away, and they follow a straight direction ; one of the most typical symptoms. Sometimes they have a sort of indescribable wrath. The furious excesses of epileptics resemble those of the maniacs ; they crush everything in their way, and whenever they strike or kill, they multiply their strokes to an incredible amount. The epileptic tears his victim piece-meal, as no body else could do. Epileptics sometimes have a pathological religiousness ; they have sacred visions, and then their hallucinatory character becomes absolutely mystical.

3. *Mental epileptics* (*larvés*).—These have eccentric characters. They have partial eclipses of intellect, with a sort of periodical course, and always do the same things in the same manner. It is, so to speak, a new photographic proof taken at each repetition of their fit. They have also automatic movements, but no peculiar accident of the body. This has been called mental epilepsy, by the Americans. The manifestation is wholly mental ; it is a disease of the intellect. They do presumptuous actions ; they abandon their position, change religion, take strange resolutions. If they remain long under observation, one will notice the periodicity of these fits. All this disappears under the use of bromide of potassium. This is a partial disease, the same as some eruptive affections without eruption, *variola sine variolis.* Crimes committed without any motive essentially belong to epilepsy.

Hippocrates, referring to *morbus sacer*, speaks of strange patients, having no fit, yet rushing out of their beds and running out at night. It seems from this that he knew mental epilepsy. Lasègne and Legrand du Saulle came across a striking example some years ago. A divorce *a toro* was asked for by a woman who complained that her husband (at night) would rise without any cause,* and would run out bare-headed and bare-footed even if it was freezing cold. Once he was away for eighteen months ; another time he went aboard a ship at Havre, and found out what

* Le Praticien, 18 Oct., 1880.

he had been doing after reaching Bombay. This man, when questioned about his actions could not tell how they could happen so. It was evidently mental epilepsy; besides these singular impulses, his manners had nothing odd nor remarkable.

SIMULATIONS OF VENEREAL OUTRAGES ON YOUNG CHILDREN. (*L'Union Méd.*, *Nov.* 6, 1880.)

A great deal of interest was manifested some time ago, in a paper read before the Academy of Medicine of Paris, by A. Fournier, with the above title. The author describes how he succeeded in exacting the truth from a little girl, and saved the reputation of an innocent victim. He recapitulates thus his considerations:

1. A certain number of facts exist, to which we may give the collective name of *simulation of criminal outrages on young children of the feminine sex.* These facts summarily consist in this: an artificial production on a little girl of lesions of the vulva which should resemble those of an outrage; and imputation of such an outrage to an imaginary author, for the benefit of the simulator.

2. Clinically, it is not impossible for these *artificial* lesions to betray themselves through some peculiarity, or some local incident. But this would be a fortuitous case; and, in principle as well as in practice, we know of no clinical symptom sufficient to differentiate with certainty an inflammation of the vulva occasioned by friction, from one resulting from a criminal outrage.

3. In cases of this class, the discovery of simulation will not depend so much on clinical phenomena as on other signs foreign to the medical art; the attitude, answers, hesitations, contradictions of the child, the antecedents of the simulator, various circumstances of the cause, etc.

4. That in case the physician, even while discharging his duty, should happen to discover the truth, he has more than a right, but it is a duty for him to overthrow a criminal accusation, and preserve the honor, the liberty, and the interests of an innocent individual.

5. It is important for the security of everybody, and to preserve the dignity of the profession, that in such affairs, the physician should issue certificates stating the lesions observed only when required to do so by a competent authority whose duty it is to get them ; and it is not less important that, in that kind of certificates, the physician should confine himself to the description of the lesions observed, without venturing into the etiological interpretation of these lesions, which interpretation can hardly ever be inferred from the symptoms.

6. Various moral motives serve to inspire simulators in these cases. One of the most general is a monetary speculation, which is called *chantage au viol*, (black-mail) appropriately.

7. Lastly, inflammations of the vulva of various origins, most always spontaneous, have often been the basis of accusation of outrages; and it is not unusual that these illegitimate imputations have seemed justified, either by the unconscious answers of sham victims, or even by the false depositions of prematurely corrupted children.

TREATMENT OF WHOOPING COUGH BY THE INHALATION OF SPIRITS OF TURPENTINE. By Dr. Barèty (of Nice). (*L'Union Méd.*, *Nov.* 4, 1880.)

About four years ago, I had to treat three children suffering from whooping cough in the same family. I was treating them with the common remedies, emetics, extr. of belladonna, syrup of codeia, etc., but without any marked result, when at the time when the spasms recur most frequently, I had an opportunity to make a most interesting discovery.

One of the children, precisely the one most severely attacked, was put to bed in a room recently painted, which gave a strong odor of oil of turpentine. From that time it happened that the crises became less intense and less fatiguing, and that the disease was much shorter in this case than in the others.

This occurrence impressed me vividly, and I did not doubt that the rapid improvement must be referred to the oil of turpentine which, escaping from the fresh paint, pervaded the atmosphere of the room and was breathed by the young patient. Accordingly I resolved in the future to try inhalation of spirits of turpentine. This I had opportunities to do many times successfully.

Here is my *modus operandi:* I fill two deep plates half full with spirits of turpentine; I place one of them under the bed and the other in a corner of the room.

The child, or children, sleep in that room saturated with vapors of the spirits of turpentine, and spend a part of the day in it.

The spirit is renewed every time it is expedient. The air is renewed in the room once or twice a day.

The crises of spasms rapidly diminish, the disease takes a very mild character and hardly lasts a month on the average.

---

SALT AS A PROPHYLACTIC IN DIPHTHERIA.—In a paper read at the Medical Society of Victoria, and published in the *Australian Medical Journal* for June, 1880, "On the Free Use of Salt as a Prophylactic against Diphtheria," Dr. Day stated that, having for many years past looked upon diphtheria in its early stage as a purely local affection, characterized by a marked tendency to take on putrefactive decomposition, he has trusted most to the free and constant applications of antiseptics; and when their employment has been adopted from the first and has been combined with judicious alimentation, he has seldom seen blood poisoning ensue. In consequence of the great power which salt possesses in preventing the putrefactive decomposition of meat and other organic matter, Dr. Day has often prescribed for diphtheritic patients living far away from medical aid the frequent use of a gargle composed of a tablespoonful or more of salt, dissolved in a tumbler of water; giving children who cannot gargle a teaspoonful or two to drink occasionally. During the prevalence of diphtheria he recommends its use instead of sugar in the food of children, adults using the gargle as a prophylactic, three or four times a day.—*Medical and Surgical Reporter.*

